# An Exploratory Approach to Analyzing Alcohol Control Policy Opinions Held by Ontario Adults

**DOI:** 10.3390/ijerph7030827

**Published:** 2010-03-08

**Authors:** Anca R Ialomiteanu, Norman Giesbrecht, Edward M Adlaf, Hyacinth Irving, Angela Paglia-Boak, Jürgen Rehm

**Affiliations:** 1 Centre for Addiction and Mental Health, 33 Russell Street, Toronto, Ontario M5S 2S1, Canada; E-Mails: anca_ialomiteanu@camh.net (A.R.I.); Edward_adlaf@camh.net (E.M.A.); hm.irving@utoronto.ca (H.I.); angela_boak@camh.net (A. P.-B.); jtrehm@aol.com (J.R.); 2 Dalla Lana School of Public Health, University of Toronto, 155 College Street, Toronto, Ontario, M5T 3M7, Canada; 3 Department of Psychiatry, Faculty of Medicine, University of Toronto, 250 College Street, Toronto, Ontario, M5T 1R8 Canada; 4 Technische Universität, Dresden, Pressetelle 01062, Dresden, Germany

**Keywords:** alcohol policy opinions, latent class analysis, Ontario adults, survey

## Abstract

Telephone interview data from a representative sample of 1,216 Ontario adults were analyzed using latent class analysis to determine whether distinct and homogeneous classes of individuals could be identified based on their responding patterns to 11 alcohol policy items. Five latent classes were identified and labeled as: dedicated liberalizers, moderate liberalizers, moderate controllers, dedicated controllers, and an ambivalent class. Multinomial regression analysis indicated that demographic and alcohol factors differentiated the classes. Those most opposed to alcohol controls, dedicated liberalizers, were more likely to be male, younger and heavier drinkers. Given their young age it is possible that further erosion of public support for alcohol controls may be expected.

## 1. Introduction

Alcohol policy is an essential component of population-level initiatives to reduce drinking-related damage [1,2]. Various social and economic factors have influenced political initiatives and decisions involving alcohol policy [3], including for example, ideology of policy-makers and managers, perceived level of damage from alcohol and public health or alcohol industry advocacy on alcohol issues [4]. Alcohol policies are also shaped by public opinion about alcohol and about the role of government in regulating individual behaviours [4]. Therefore, an analysis of public opinion on alcohol policy is relevant to understanding the impact of research findings on knowledge translation in the political arena, and the policy development process [5].

There is substantial literature on public opinion on alcohol policy, including studies going back several decades and involving several jurisdictions [6,7]. While there are initiatives to organize policies into conceptual schemes, such as alcohol access, promotional activities, counter promotion and public information, and intervention [7], in general, the structure and selection of policies to study appears to be driven more by practical considerations such as, what are current or recent policies activities, rather than by theoretical models. Nevertheless, the list of policy opinion questions used in a 1989 Canadian study [8] provided a snap-shot of a range of interventions used in many jurisdictions, and became part of the core questions used in a number of subsequent studies [7,9].

However, the relationship between public opinion and the implementation of alcohol policy measures is complex. A change in policy may bring public opinion along with it. At other times, public opinion may remain unshakable despite a policy change (e.g., [6]). Studies of public opinion typically found stronger public support for interventions least intrusive such as information and persuasion campaigns or warning labels, and modest public support for regulations that control access to alcohol and potentially affect all consumers, such as higher taxes, lower outlet density or shorter hours of sale [7–10].

The vast majority of public opinion research on alcohol policy, typically based on single-item measures, has centered on establishing the level of support for policy initiatives, its correlates and trends [7,10–17]. Although some research has assessed the dimensional aspects of alcohol policy control opinions held by the general public using factor analysis techniques [14,18–20], few have assessed individual-level responding patterns. Using latent class analysis (LCA), we describe the responding patterns to 11 alcohol policy opinion items asked of adults in a general population survey in Ontario, the most populous Canadian province.

This paper addresses the following research question: is it possible to identify a number of homogenous classes of individuals that display similar responding patterns among a set of alcohol policy opinion items, assuming that a latent (*i.e.*, unobserved) class structure underlies the relations among the observed variables? Identification of classes of individuals on alcohol policy opinions is relevant to understanding the dynamics of the alcohol policy development process—for example, which sectors of the population that policy entrepreneurs, alcohol industry or public health advocates—focus their attention on. It is also relevant to developing a basis for assessing the relative stability of support or opposition to alcohol policies.

## 2. Methods

### 2.1. Data

Our analysis was based on telephone interviews with 1,216 respondents derived from the 2005 cycle of the CAMH Monitor, a repeated monthly cross-sectional telephone survey of adults (18 years or older) in Ontario, Canada, conducted by the Centre for Addiction and Mental Health and administered by the Institute for Social Research, York University. This general population survey is part of a long-standing series of annual cross sectional surveys conducted by the Centre of Addiction and Mental Health on a range of topics with foci on tobacco, alcohol, illicit drugs, perscription drugs, gambling and mental health issues. Each year approximately a dozen questions are set aside to obtain public opinion of Ontarians on selected alcohol policy topics. The selection of the alcohol policy questions for each annual survey is based on two main factors: (1) a bi-annual or tri-annual repetition of ‘core’ questions so that monitoring changes in public opinion over time is feasible, and (2) identification of emerging or current issues and selection or development of several questions that are considered most relevant to this topic.

Each monthly cycle of the survey employs a two-stage probability (household, respondent) selection procedure using random-digit-dialing methods and Computer Assisted Telephone Interviewing. To increase the precision of estimates within different areas of the province, the sample was equally allocated among six strata according to area code and the corresponding counties. Within each regional stratum a random sample of telephone numbers was selected with equal probability in the first stage of selection (*i.e.*, households). Within selected households, one respondent age 18 or older was selected according to the most recent birthday of household members. A minimum of 12 call-backs were placed to unanswered numbers and all households who refused to participate on the first contact are re-contacted in order to secure maximum participation. The overall response rate of the 2005 cycle of the survey was 61%. To strengthen the confidence in these data and to ensure that characteristics of the sample were similar to the Ontario population, post-stratification adjustment weights were applied according to the age, sex and regional distribution of the Ontario population (approximately 9,120,000 adults). (See [21] for sampling design details.) The final weights used for the sample were a function of the sampling weight and a post stratification adjustment. Although this procedure does not remove all biases, it does provide a simultaneous adjustment for non-response and non-coverage of households without telephone [22]. Although each yearly cycle consists of 12 independent monthly samples with about 200 completions each, the alcohol policy items were included only in the 6-month period from July to December 2005.

Demographic characteristics of the sample were as follows: 53% were female, the mean age was 45 years (SD = 17.2), 65% were married, 64% had post secondary education (mean years of education is 13.8 years, SD = 2.6), 56% reported an income of $CAD 50,000 or higher, and 76% were past-year drinkers. All cycles of the CAMH Monitor have been approved by the Research Ethics Boards of the Centre for Addiction and Mental Health and York University.

### 2.2. LCA Variables

The following 11 alcohol policy opinion items were included in the LCA: (1) alcoholic beverages should have warning labels (yes or no); (2) the government should prohibit wine, liquor and beer advertising on TV (yes or no); (3) the government should prohibit wine, liquor and beer companies from sponsoring sporting or cultural events (yes or no); (4) taxes on alcoholic beverages should be increased, decreased or remain the same; (5) hours of alcohol sales in restaurants, bars or taverns should be increased, decreased or remain the same; (6) efforts to prevent drunken customers at bars, restaurants or taverns to be served should be increased, decreased or remain the same; (7) the legal drinking age should be raised, lowered, remain the same; (8) the number of Liquor Control Board of Ontario (LCBO) stores is too few, too many, or about right; (9) the number of beer stores is too few, too many, or about right; (10) governments should be required to consult with health experts before making legislation or policy changes to the way alcohol is sold (strongly agree, somewhat agree, somewhat disagree, strongly disagree); and (11) the Ontario government should close all LCBO stores, and allow privately-run stores to sell alcohol (strongly agree, somewhat agree, somewhat disagree, strongly disagree). All 11 questions also had a “don’t know” response option.

Questions 1 to 7 focus on counter-promotion and public information (1,2), control of promotion (3), alcohol access (4,5) and interventions (6,7). An underlying rationale of questions 8 to 11 was the tentative initiatives by the Ontario government to increase access to alcohol and possibly privatization of the alcohol retailing distribution system, deliberations that were underway at the time of this survey.

### 2.3. Independent Variables

Demographic factors known to be related to alcohol policy opinions [7,12,13] were employed in our analysis. These included sex, age, marital status, years of education, and household income. Alcohol consumption measures included: (1) a drinking status variable with three values: past year drinker, former drinker, and lifetime abstainer, (2) the average number of drinks consumed per week (estimated using the usual quantity by usual frequency approach) and (3) the Alcohol Use Disorders Identification Test (AUDIT), designed to detect problem drinkers at the less severe end of the spectrum of alcohol problems [23,24]. We used the standard cut-off score of 8 or more out of 40 as an indication of problem drinking (range 0−33, mean 3.27).

### 2.4. Analyses

We used LCA to examine the classes of responding patterns to 11 policy items. Respondents were assigned to classes based on their posterior probabilities for class membership for a particular responding pattern. The 11 policy items were modeled as indicators of a categorical latent variable. A series of unrestricted models (2- to 6-class) were fit to the data using Mplus version 4.2 [25] with maximum likelihood ratio (MLR) estimation. MLR uses random starting values to optimize the parameters. In the initial stage, 500 random sets of starting values were used and a maximum of 2,000 iterations were allowed for each initial stage. In the final stage, 20 optimizations were employed. We began with a 2-class model and proceeded stepwise until the model did not improve further.

To accommodate the complex survey design, the LCA was performed accounting for the sampling weights. To determine the fewest number of classes, several model-fit statistics were examined: the sample size adjusted Bayesian information criterion (ABIC), the Akaike information criteria (AIC), entropy, and the Vuong-Lo-Mendell-Rubin Likelihood Ratio Test (VLMR LRT), which is a test of fit between the model of interest and a model with one less class. Despite the availability of multiple fit statistics, the decision of the most appropriate solution is a function of both statistical and substantive reasoning.

In order to identify socio-demographic characteristics that discriminate among the 5 classes, we employed multinomial logistic regression using independent variables shown to be related to opinions regarding alcohol controls—sex, age, years of education, household income category and AUDIT score. The missing value loss for the multinomial regression due to listwise deletion suggested a negligible impact given that the amount of loss was minimal (91.9% or 1,118 respondents were retained).

To further analyze whether there is a single dimension underlying the LCA results, we conducted a confirmatory factor analysis and constructed a scale based on all items with a factor loading greater than 0.4. Higher values on this scale indicated increased support for alcohol policy control. These analyses were conducted using *Stata* 9.0, which allowed us to account for the complex survey design.

## 3. Results

### 3.1. Class Membership

Table 1 shows results of the 2- to 6-class solutions. Although the VLMR test indicated that the 2-class solution showed the best fit, the BIC values were the lowest (25,260.8) and the entropy values were the highest (0.83) for the 5-class solution. Moreover, the item profiles of the 5-class solution also provided a more interpretable class structure. The mean class probabilities, which indicate the probability of cases being correctly assigned to the class, were 0.944, 0.931, 0.863, 0.890 and 0.930 for class 1 to 5, respectively.

Table 2 shows characteristics of the 5 classes according to the 11 policy items. We have labeled the 5 classes as follows: (1) dedicated liberalizers (class 1; n = 102; 9.5%); (2) moderate liberalizers (class 3; n = 415; 35.9%); (3) moderate controllers (class 4; n = 437; 34.7%); (4) dedicated controllers (class 2; n = 127; 10.3%); and ambivalent (class 5; n = 135; 9.7%).

The dedicated liberalizer and the moderate liberalizer classes represent responding patterns of greater liberalization. Beginning with the “dedicated liberalizer” class, we see that this group, which represents 9.5% of adults, holds strong opposition to alcohol controls: 85.6% believe that the government should not prohibit alcohol advertising on television; 92.1% believe that the government should not prohibit alcohol companies from sponsoring sporting events; and 38.6% believe that taxes on alcohol should be decreased. This class also shows the strongest support for increased access to alcohol: 37.6% indicated that hours of alcohol sales should be increased; 15.6% responded that the legal drinking age should be lowered; 69.0% indicated that there should be more LCBO stores, and 79.9% indicated that there should be more beer stores. This class also shows the highest support for privatizing LCBO stores (33.5%).

The larger “moderate liberalizer” class, representing 35.9% of adults, generally displays a responding pattern similar to the dedicated liberalizer class; however, they are more likely to adopt status-quo positions. Most notably, compared to the dedicated liberalizers, moderate liberalizers are more likely to indicate that taxes should remain the same (65.8% *vs*. 47.9%); hours of sale should remain the same (82.6% *vs*. 59.5%); the number of LCBO stores should remain the same (92.9% *vs*. 27.9%); the number of beer stores should remain the same (96.6% *vs*. 17.5%). The moderate liberalizer class also departs from the dedicated liberalizers class by showing greater support for raising the legal drinking age (14.7% *vs*. 6.7%), greater support for increasing efforts to prevent drunken customers from driving (65.5% *vs*. 46.3%), and lower support for increasing the number of LCBO stores (3.1% *vs*. 69.0%).

The dedicated controller and moderate controller classes represent responding patterns of greater support for alcohol controls. Beginning with the “dedicated controller” class, which represents 10.3% of adults, we see that his group strongly supports alcohol controls: 98.8% approve of warning labels; 79.4% agree that the government should prohibit alcohol advertisement on television; 72.8% agree that the government should prohibit alcohol companies from sponsoring sporting events; 67.5% indicate that taxes should be increased; 48.7% indicate that hours of sale should be decreased. This class also displays the strongest support for raising the legal drinking age (68.1%), reducing the number of LCBO stores (50.5%) and beer stores (55.4%).

The larger “moderate controller” class, which represents 34.7% of adults, displays a responding pattern similar to the dedicated controllers with the following exceptions. First, they show weaker support for warning labels (80.6% *vs*. 98.8%), the government prohibition of alcohol advertisements on television (61.2% *vs*. 79.4%) and sponsorship of sporting events by alcohol companies (40.2% *vs*. 72.8%). Second, they are more likely than the dedicated controller class to adopt status quo positions: more indicate that taxes should remain the same (71.1% *vs*. 17.5%); that hours of sales should remain the same (78.4% *vs*. 23.3%); that the legal drinking age should remain the same (51.2% *vs*. 15.0%), and that efforts to prevent drunken customers from being served should remain the same (17.4% *vs*. 3.8%). In addition, this class is more likely than the dedicated controllers to support the status quo of alcohol access. More indicate that the number of LCBO stores should remain the same (96.1% *vs*. 20.1%) and that the number of beer stores should remain the same (96.3% *vs*. 21.1%).

The “ambivalent” class, representing 9.7% of adults, captures a responding pattern representative of those with ambivalent, uninformed or ephemeral opinions about alcohol policy. By far, the most distinguishing feature of this class is the high rates of don’t know responses, which range from 8.2% to 65.3% (median = 36.7%). Also, there is some suggestion that they are less knowledgeable about alcohol policy given that 34.4% indicate that they do not know the legal drinking age.

### 3.2. Discriminators of the Five Classes

Table 3 shows the demographic and alcohol consumption characteristics of the 5 classes and Table 4 shows results of the multinomial logit model predicting class membership. First, in Table 4, we see that with the exception of years of education, all independent variables (sex, age, income category and AUDIT score) are significantly related to class membership, after adjusting for all other variables. Given that recent research has shown long-term increases in liberalizing attitudes in both Canada and the United States [9,26], we chose the dedicated liberalizers to serve as the reference group in the regression analysis. Thus, each of the four columns in Table 4 provides odds ratios associated with each demographic and alcohol factor for the given latent class relative to the dedicated liberalizers.

As seen in Table 4, compared to the dedicated liberalizer class, those in the remaining four latent classes were less likely to be male and had lower AUDIT scores. Respondents in the ambivalent, moderate and dedicated controller classes were older than those in the dedicated liberalizer class. The odds ratios associated with household income showed that, relative to dedicated liberalizers, respondents in the ambivalent and dedicated controller classes were more likely to reside in households with incomes of less than $30,000. Education was unrelated to latent class membership.

### 3.3. Results from the Confirmatory Factor Analysis

Finally, all respondents were also rank-ordered on the composite unidimensional scale (measuring levels of support for more liberalization in alcohol policy) resulting from the confirmatory factor analysis, which showed sufficiently high goodness of fit values to indicate that the assumption of unidimensionality corresponded well to the data (Comparative Fit Index: 0.93; root mean square error of approximation <0.08; other details not shown). This scale was a composite of all 11 alcohol policy measures, with higher values indicating stronger support for alcohol controls. The five latent classes were also rank-ordered in terms of support of control, with the “Ambivalent” class treated as neutral. A polychoric correlation between the ranked five classes and the scale showed a coefficient of 0.864 (SE = 0.012). This suggests that one single dimension seems to be underlying the five LCA classes.

## 4. Discussion

We found that our 11 policy items could be used to group people into five distinct and homogeneous classes. Still, we must recognize that our paper has several limitations. It is feasible that the four main substantive classes—from dedicated liberalizers to dedicated controllers are largely degrees on one continuum from those wishing liberalization of alcohol controls to those favouring more control. Second, our data are based on unvalidated self-reports. Third, we are lacking data on opinions among non-responders. Fourth, in the absence of replication, the stability of our LCA solution is unknown, and indeed, alternative solutions are possible. Moreover, our LCA solution might be regionally unique, given that several of our policy items are of a regional nature.

Nonetheless, in our view, this study has made several contributions. First, it illustrates the population distributions of alcohol control opinions. It is notable that liberalizers and controllers are equally distributed, suggesting a potential tension surrounding alcohol issues. The second contribution of our study is that it suggests that alcohol policy opinions are not fully dimensional in character. Although the four classes of dedicated liberalizers to dedicated controllers appear to exist on a continuum, the presence of the ambivalent class suggests a subtype character of public opinion. The demographic profile of the ambivalent class shows that they tend to be older and less educated although, unlike the existing literature on don’t know and no opinion responders [27], these effects did not hold in the multivariable analysis.

As noted above, these data from a 2005 survey of Ontarians is part of a series of annual cross-sectional surveys, which address a number of topics, including a small set of items reserved for alcohol policies. The list of questions is not identical from year to year. However, a previous publication [12] in which temporal trends in responses to identical questions were analyzed, demonstrated a similar variation in support by type of policy topic and a very gradual decline in support for control measures between 1989 and 1998. Nevertheless some questions typically get strong support from year to year (e.g., introduction of warning labels, interventions to cut of service to drunken customers) and others only from a minority (e.g., increase in taxes on alcohol or reduction of number of outlets that sell alcohol) [6–9,12].

Our findings have several implications. First, the summation of policy items may not fully capture the underlying heterogeneity of responding patterns. Moreover, the simple percentage support includes ambivalent/non-attitude responders, whose impact of support estimates may involve complex mechanisms [28]. Indeed, to what extent opinions are stable in this group is unknown. Second, we need to be cautious about the interpretation of the so-called “status quo” positions, given that our data suggest two independent classes—moderate liberalizers and moderate controllers—that both represent this response. Furthermore, there may be substantial variation among respondents, including those who support a status quo position, in the accuracy of their knowledge about alcohol policy.

Third, one group of particular interest is the dedicated liberalizers, who hold the strongest views on loosening controls, but more importantly may be a demographic that will increase in impact on alcohol policies in coming years given their typical young age, and current higher average incomes. It is possible that this group would be of special interest to alcohol producers, advertisers and retailing networks. Will their views become more control-oriented with increasing age, or do these findings point to a birth cohort with strong views on increasing access to alcohol combined with heavier drinking? Our data do not allow us to empirically explore these questions. If their views remain with increasing age, what are the implications for alcohol policy and public health?

Fourth, in these studies the underlying reasons for the public opinion held by the respondents is a largely underdeveloped arena and might be a worthy topic for future research. We do not know at this stage whether the perceived impact of a policy on self, significant others or the community are important dimensions influencing the views of respondents. Or, to what extent alcohol policy perspectives might be influenced by general political orientations to government roles, and/or personal experience with damage and disruption due to heavy drinking by others, or positive experiences associated with drinking occasions including heavy drinking.

It has been noted by Kaskutas [10] that respondents are more likely to support policies that are least intrusive from their perspective (e.g., warning labels, server intervention, education) and less supportive of those that restrict access (e.g., higher taxes on alcohol, lower density, shorter hours). While a conceptual framework with regard to ‘intrusiveness’ has not been developed, several dimensions are offered for future consideration and elaboration: (a) demographic scope of the intervention—impacting all drinkers *vs*. only a minority; (b) temporal and geographic scope of the intervention—impacting all drinking occasions *vs*. only a minority; (c) potency of the intervention—whether it has mandatory versus voluntary implications. Alcohol-related taxes might be considered of wide scope—potentially impacting all drinkers, all occasions, have a mandatory dimension and therefore potentially intrusive, even if the actual impact on the low volume drinkers is minimal. In contrast, while a server intervention may have a mandatory dimension (*i.e.*, a bartender can cut off service), it likely only applies to the minority of drinking occasions and drinkers and therefore likely not considered intrusive by most respondents.

In general, knowledge about the conceptual underpinnings of which policies are supported or rejected by whom is at a preliminary stage, and this area would benefit from further work. From a methodological point of view, it may be sufficient to measure alcohol policy opinions with fewer items since we found only one underlying dimension.

Finally, this cross-sectional analysis of recent Ontario data provides a basis for future initiatives. Recent trends in actual policy, overall drinking and media attention to alcohol issues and alcohol problems may also play a role in the results reported here. It would be of interest to see if similar classes emerged in studies of alcohol management in various jurisdictions and environments. Such studies might use this method and contrast the findings from a jurisdiction where alcohol is widely and easily available compared to one where access was more strictly controlled, or compare jurisdictions with a dramatic increase (or decline) in alcohol control initiatives.

## Figures and Tables

**Table 1.** LCA Model Fit.

**Model**	**BIC**	**Adjusted BIC**	**AIC**	**# Parameters**	**Entropy**	**VLMR LRT**	**P value**	**Test K-1 classes**
2 Classes	26030.1	25817.2	25688.1	67	0.81	–15376.6	0.001	1 (Ho) *vs*. 2 classes
3 Classes	25544.9	25224.1	25029.5	101	0.78	–12777.1	0.979	2 (Ho) *vs*. 3 classes
4 Classes	25325.1	24896.3	24636.2	135	0.81	–12413.7	1.000	3 (Ho) *vs*. 4 classes
5 Classes	25260.8	24723.9	24398.3	169	0.83	–12030.1	1.000	4 (Ho) *vs*. 5 classes
6 Classes	25353.1	24708.3	24317.1	203	0.81	–11955.6	1.000	5 (Ho) *vs*. 6 classes

Notes: (1) using complex survey design; (2) VLMR LRT = Vo-Lo-Mendell-Rubin Adjusted Likelihood Ratio Test.

**Table 2.** Conditional Probabilities (%) of Opinions on Alcohol Controls for the 5-Class Model.

**Alcohol Policy Questions**	**Dedicated Liberalizers**	**Moderate Liberalizers**	**Ambivalent**	**Moderate Controllers**	**Dedicated Controllers**

(N = 1216)	(n1 = 102; 9.5%)	(n3 = 415; 35.9%)	(n5 = 135; 9.7%)	(n4 = 437; 34.7%)	(n2 = 127; 10.3%)
**1.** Alcoholic beverages should have warning labels					
Yes	45.6	47.3	53.9	80.6	98.8
No	53.7	50.8	20.5	15.2	1.1
DK	0.7	2.0	25.6	4.2	0.1
**2.** The government should prohibit wine, liquor and beer advertising on TV					
Yes	14.4	3.9	22.1	61.2	79.4
No	85.6	91.6	48.9	33.0	15.8
DK	0	4.5	29.0	5.8	4.7
**3.** The government should prohibit wine, liquor and beer companies from sponsoring sport events					
Yes	6.3	0.5	24.1	40.2	72.8
No	92.1	99.5	45.9	54.8	16.6
DK	1.6	0	30.0	5.1	10.7
**4.** Taxes on alcoholic beverages should be					
Increased	11.8	0.9	16.7	16.6	67.5
Remain the same	47.9	65.8	32.5	71.1	17.5
Decreased	38.6	31.7	16.4	10.1	6.8
DK	1.7	1.6	34.3	2.1	8.2
**5.** Hours of alcohol beverages sales in restaurants and bars should be					
Increased	37.6	7.7	11.1	0	18.6
Remain the same	59.5	82.6	38.0	78.4	23.3
Decreased	1.6	7.4	7.7	19.6	48.7
DK	1.3	2.3	43.2	1.9	9.5
**6.** Efforts to prevent drunken customers being served should be					
Increased	46.3	65.5	46.0	67.7	71.1
Remain the same	45.3	28.7	5.8	17.4	3.8
Decreased	3.6	2.4	5.8	11.8	23.5
DK	4.8	3.4	36.9	3.2	1.6
**7.** The legal drinking age should be					
Raised	6.7	14.7	21.6	30.0	68.1
Remain the same	65.1	67.7	29.3	51.2	15.0
Lowered	15.6	6.8	6.4	2.9	2.3
Not know drinking age	12.6	10.8	34.4	15.5	13.6
DK	0	0	8.2	0.3	1.0
**8.** There should be fewer or more LCBO stores in Ontario					
Fewer	0	0.5	1.0	1.4	50.5
Remain the same	27.9	92.9	27.2	96.1	20.1
More	69.0	3.1	6.5	1.1	0
DK	3.0	3.5	65.3	1.4	29.4
**9.** There should be fewer or more beer stores in Ontario					
Fewer	1.8	1.1	5.7	2.6	55.4
Remain the same	17.5	96.6	28.2	96.3	21.1
More	79.9	0	1.0	0.6	0
DK	0.8	2.3	65.1	0.5	29.4
**10.** The government should consult health experts before making changes to the way alcohol is sold					
Strongly agree	26.5	16.4	24.8	54.9	73.5
Somewhat agree	30.6	44.8	23.1	34.2	13.8
Somewhat disagree	26.7	24.5	8.9	4.5	2.8
Strongly disagree	16.2	13.1	6.5	1.7	4.5
DK	0	1.1	36.7	4.7	5.3
**11.** The government should close all LCBO stores and allow privately-run stores to sell alcohol					
Strongly disagree	30.8	48.6	20.0	58.9	50.0
Somewhat disagree	16.2	22.1	11.3	21.5	16.8
Somewhat agree	14.1	16.5	13.0	9.5	7.7
Strongly agree	33.5	11.6	15.0	3.9	12.7
DK	5.4	1.2	40.1	6.2	12.7

**Table 3.** Demographic Characteristics and Alcohol Status According to the 5 Classes.

**Demographic Characteristics**	**Dedicated Liberalizers**	**Moderate Liberalizers**	**Ambivalent**	**Moderate Controllers**	**Dedicated Controllers**

(N = 1216)	(n1 = 102; 9.5%)	(n3 = 415; 35.9%)	(n5 = 135; 9.7%)	(n4 = 437; 34.7%)	(n2 = 127; 10.3%)
**Gender**			***		
% Males	78.6	56.5	32.1	36.4	32.4
**Age**			**		
18−29	26.6	27.5	5.6	17.8	16.2
30−39	25.6	20.0	14.4	21.5	20.2
40−49	22.2	24.5	15.9	24.0	19.8
50−64	20.4	16.3	26.9	19.6	23.2
65+	5.2	11.7	37.2	17.1	20.6
Mean age	40.5	42.1	56.7	46.1	47.3
SE	1.91	0.93	2.17	1.02	1.89
**Marital status**			**		
Married	53.6	66.5	61.3	66.3	72.2
Previously married	11.5	10.9	13.3	14.2	11.7
Never married	34.9	22.6	16.0	19.5	16.1
**Education**			**		
<High school	8.8	7.1	23.2	12.4	11.2
Completed High school	27.5	28.6	23.5	24.2	17.7
Some post-secondary	35.9	38.5	28.4	30.7	29.6
University	27.8	25.8	24.9	32.7	41.5
Mean Years of Education	13.9	13.8	12.8	13.7	14.3
SE	0.26	0.12	0.32	0.18	0.27
**Household Income**			***		
<$30,000	6.6	5.2	23.3	11.1	19.1
$30,000−$49,000	15.9	15.6	15.9	13.3	18.9
$50,000−$79,000	13.6	22.6	11.3	22.6	19.2
$80,000+	45.7	39.5	18.9	37.7	21.6
DK/REF	18.2	17.1	30.7	15.3	21.3
**Drinking Status (past-year)**			***		
Past year drinker	89.7	90.9	49.3	78.1	33.2
Former drinker	8.1	6.7	27.9	17.5	26.2
Abstainer	2.2	2.4	22.7	4.4	40.6
**Average Number of Drinks/Week**^a^			***		
Mean No. of Drinks	7.08	3.93	1.45	2.08	0.85
SE	0.99	0.34	0.36	0.23	0.43
**AUDIT 8**+			***		
% yes	29.1	11.8	1.7	6.5	1.0
Mean AUDIT score	6.23	4.09	1.31	2.78	1.10
SE	0.67	0.20	0.20	0.16	0.30

Note: significance chi-square:

** p < 0.01;

*** p <0.001;

a Derived from the product of usual quantity by usual frequency of alcohol intake in the past 12 months.

**Table 4.** Multinomial logistic regression results (OR) predicting class membership (N = 1,118).

	***Dedicated Liberalizers vs:***				
**Demographic Characteristics**	**Moderate Liberalizers**	**Ambivalent**	**Moderate Controllers**	**Dedicated Controllers**	Overall Significance

	OR	OR	OR	OR	
		
**Male**	0.426**	0.166***	0.185***	0.198***	***
**Age**	1.005	1.044***	1.017*	1.020*	***
**Education (years)**	0.976	0.932	0.968	1.087	NS
**Household Income** (>$80,000–reference group)					***
<$30,000	0.708	4.863*	1.724	4.290*	
$30,000–$79,000	1.527	1.371	1.259	2.223	
DK/Refused	0.804	1.247	0.503	1.115	
**AUDIT** score	0.905*	0.649***	0.811***	0.538**	***

Note: OR = odds ratio; comparison group is “dedicated liberalizers”;

Wald test: * p < 0.05,

** p < 0.01,

*** p < 0.001.
